# Synaptic Plasticity on Motoneurons After Axotomy: A Necessary Change in Paradigm

**DOI:** 10.3389/fnmol.2020.00068

**Published:** 2020-04-30

**Authors:** Francisco J. Alvarez, Travis M. Rotterman, Erica T. Akhter, Alicia R. Lane, Arthur W. English, Timothy C. Cope

**Affiliations:** ^1^Department of Physiology, Emory University School of Medicine, Atlanta, GA, United States; ^2^Department of Biomedical Engineering, School of Biological Sciences, Georgia Institute of Technology, Atlanta, GA, United States; ^3^Department of Cellular Biology, Emory University School of Medicine, Atlanta, GA, United States

**Keywords:** motoneuron, axotomy, regeneration, synaptic plasticity, microglia, astrocytes, Ia afferent synapses, sensorimotor integration

## Abstract

Motoneurons axotomized by peripheral nerve injuries experience profound changes in their synaptic inputs that are associated with a neuroinflammatory response that includes local microglia and astrocytes. This reaction is conserved across different types of motoneurons, injuries, and species, but also displays many unique features in each particular case. These reactions have been amply studied, but there is still a lack of knowledge on their functional significance and mechanisms. In this review article, we compiled data from many different fields to generate a comprehensive conceptual framework to best interpret past data and spawn new hypotheses and research. We propose that synaptic plasticity around axotomized motoneurons should be divided into two distinct processes. First, a rapid cell-autonomous, microglia-independent shedding of synapses from motoneuron cell bodies and proximal dendrites that is reversible after muscle reinnervation. Second, a slower mechanism that is microglia-dependent and permanently alters spinal cord circuitry by fully eliminating from the ventral horn the axon collaterals of peripherally injured and regenerating sensory Ia afferent proprioceptors. This removes this input from cell bodies and throughout the dendritic tree of axotomized motoneurons as well as from many other spinal neurons, thus reconfiguring ventral horn motor circuitries to function after regeneration without direct sensory feedback from muscle. This process is modulated by injury severity, suggesting a correlation with poor regeneration specificity due to sensory and motor axons targeting errors in the periphery that likely render Ia afferent connectivity in the ventral horn nonadaptive. In contrast, reversible synaptic changes on the cell bodies occur only while motoneurons are regenerating. This cell-autonomous process displays unique features according to motoneuron type and modulation by local microglia and astrocytes and generally results in a transient reduction of fast synaptic activity that is probably replaced by embryonic-like slow GABA depolarizations, proposed to relate to regenerative mechanisms.

## Introduction

Peripheral nerve injuries are widely used to study neuronal responses to physical damage and axotomy, as well as the induction of regeneration programs without confounding effects of direct injury to the surrounding CNS or complex neuropathology. Motor and sensory axons injured in peripheral nerves are disconnected from their targets but can regenerate through complex programs initiated in their cell bodies, located in the spinal cord and dorsal root ganglia respectively. However, despite regeneration many patients experience long-term motor dysfunction (reviewed in Lundborg, 2003; Brushart, 2011). Poor outcomes are often attributed to the slow pace of regeneration and incorrect targeting during regeneration (reviewed in Allodi et al., 2012; Gordon and English, 2016). Most developmental axon guidance cues are not present in the adult and regenerating axons can enter nerve fascicles directing them to the wrong muscles or even tissues. These errors scramble the original connectivity of motoneurons and proprioceptors causing functional deficiencies. On the other hand, the slow speed of axon growth frequently implies long-term muscle denervation inducing muscle fiber atrophy that can become irreversible with time. Moreover, the regeneration capacity of motoneurons decreases with time after injury (Fu and Gordon, 1995). Not surprisingly much work focused on advancing microsurgery techniques for nerve repair and on facilitating regeneration and accelerating axon growth with bioengineering solutions and pharmacological and rehabilitative manipulations (reviewed in Gordon and English, 2016; Gordon, 2016; Panagopoulos et al., 2017; Tajdaran et al., 2019). But in addition to regeneration mechanisms in the periphery, it is important to also consider changes in the CNS induced by nerve injuries (reviewed in Navarro et al., 2007). After axotomy, motoneurons undergo early and late changes in gene expression that switch them to a regenerative phenotype (reviewed in Gordon, 2016). These are paralleled by structural modifications in cell bodies and dendrites (chromatolytic reaction) as motoneurons shift cellular metabolism and protein synthesis towards producing materials for axon growth and regeneration (Lieberman, 1971; Gordon, 2016). One intriguing aspect of this response is the intense shedding of synapses, particularly those of glutamatergic origin, from motoneurons after axotomy and undergoing regeneration. The significance of this plasticity is yet unclear and is the focus of this review article.

Despite a wealth of studies on synaptic plasticity around axotomized motoneurons, a coherent comprehensive view of its significance and mechanisms is yet to be established. New evidence suggests the need to reconsider three significant ideas that have led to much experimentation and data interpretation in the past. First, the assumption that synaptic plasticity around axotomized motoneurons, usually referred to as “synaptic stripping,” is a single phenomenon. There is now enough evidence suggesting that different synapses (excitatory or inhibitory, arising from injured peripheral sensory afferents or uninjured CNS neurons) undergo plastic changes that differ in mechanism, time-course, significance for regenerative processes, and functional implications after regeneration. Second, the assumption that synapse withdrawal is necessary for motoneurons entering an electrically silent state that favors regeneration. Current evidence, reviewed below, suggests this is not the case. Efficient manipulations to enhance regeneration include electrical stimulation and exercise, both based on increasing motoneuron activation (reviewed in Gordon and English, 2016). Third, synaptic changes on the cell body cannot be extrapolated to the whole input to the motoneuron. Synaptic inputs differ by whether they are lost from cell bodies only or also from dendrites.

We recently distinguished two types of synaptic plasticity after nerve injury (Alvarez et al., 2010, 2011; Rotterman et al., 2014, 2019). One type is the classically described transient loss of synapses that occurs specifically over the cell bodies of axotomized motoneurons affecting all types of synapses. These synaptic changes revert after motor axons reinnervate muscles and may be related to regeneration mechanisms. The second type induces a permanent change in spinal cord circuitry and affects the central synaptic arbors of axons (proprioceptive sensory or motor) injured peripherally. These synapses are lost not only over axotomized motoneurons but also on many other targets in the ventral horn and affect both cell bodies and dendritic arbors. The long-lasting loss of central synapses originating from proprioceptive and motor axons injured in the peripheral nerve likely reorganizes motor control spinal circuitries causing functional alterations after axons regenerate peripherally.

Additional confounds have been the diversity of models used to study this synaptic plasticity. Differences fall into three categories: the type of motoneuron (spinal, facial, hypoglossal, vagal…), nerve injury (different nerves, crush vs. cut, proximal vs. distal) and species (cats, rabbits, mice, rats, guinea pigs…). This diversity introduces high variance in reported results, but comparisons of similarities and differences also provide insights into mechanisms and significance. Importantly, different types of nerve injuries all result in axotomy of the motor axon and induce a regenerative program in the motoneuron; however, they drastically differ in motoneuron preservation and speed and efficiency of regeneration. The goal of this review is to organize this multiplicity of data to allow more precise interpretations of past results and more specific hypotheses moving forward.

## The History of “Synaptic Stripping” Over Motoneurons After Nerve Injury: Back to the Origins

**Figure 1:**
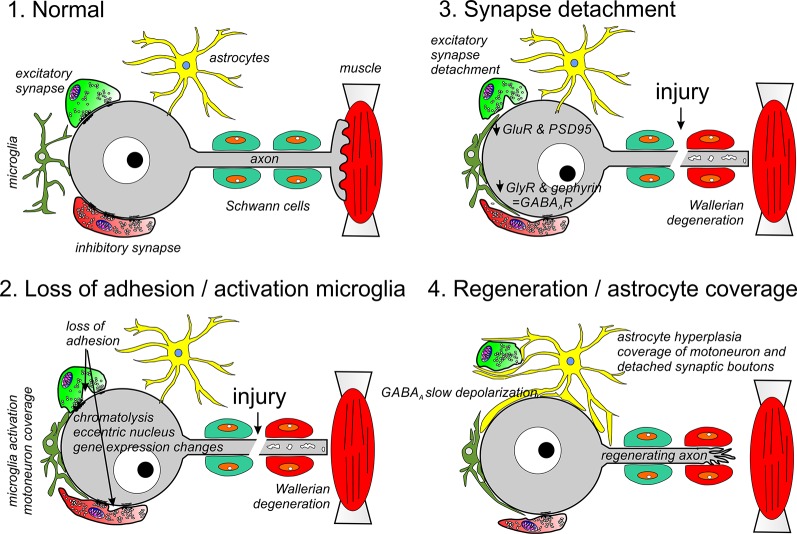
Different phases of synaptic and glia plasticity around cell bodies of axotomized motoneurons. Axotomy induces chromatolysis and rapid changes in gene expression due to positive injury signals arriving at the cell body and negative signals due to lost trophic support from muscle. During an early phase (2), adhesion in non-junctional areas is reduced and activated microglia migrate towards the cell body replacing membrane regions previously covered by synaptic bouton. In the periphery, the cut distal segment undergoes Wallerian degeneration (2, 3) and Schwann cells modify phenotype to orchestrate the removal of axon debris and upregulate trophic factors and adhesion proteins to promote regeneration. This phase is followed by reduced motoneuron expression of synaptic genes, including GlyRs and GluRs, as well as, the synaptic organizers PSD95 and gephyrin (3). This results in full detachment of many excitatory synapses and some inhibitory synapses. Finally, the motoneuron is covered by astrocytic lamellae replacing microglia (4). All changes on the surface of the motoneurons revert after the motor axon successfully reinnervates muscle: astrocyte coverage disappears, inhibitory synapses enlarge and recuperate gephyrin and GlyRs, excitatory synapses are re-established and KCC2 expression recovers resulting in the return of normal excitatory and inhibitory synaptic activity on the cell body.

Removal of synapses from the cell body of motoneurons axotomized following nerve transections was first described in an electron microscopy (EM) analysis of the rat facial nucleus published in a landmark 1968 paper (Blinzinger and Kreutzberg, 1968). In this study, the cell bodies of axotomized motoneurons were reported to lose up to 80% of their synapses and become covered by microglia. No distinction was made among different types of synapses. In some electron micrographs, microglia processes were found interposed between the cell body surface and synaptic boutons, but with no evidence of synaptic bouton degeneration or synapse phagocytosis (summarized in Figure 1). The EM images were interpreted as a “lifting” mechanism in which microglia displaced the synapses. Synapse detachment and replacement by microglia was confirmed shortly after in spinal motoneurons (Kerns and Hinsman, 1973), hypoglossal motoneurons (Hamberger et al., 1970; Sumner and Sutherland, 1973; Sumner, 1975a) and later on oculomotor motoneurons (Delgado-Garcia et al., 1988). It was found to be similar across species [mouse (Torvik and Skjorten, 1971); cat (Chen, 1978); rabbit (Hamberger et al., 1970)], including humans (Graeber et al., 1993). The term “synaptic stripping” was coined. Common among these studies was the finding that synapse losses were limited to cell bodies and proximal dendrites (Delgado-Garcia et al., 1988; Linda et al., 1992; Brännström and Kellerth, 1998) and that synapses are recovered after the motoneurons reinnervate muscle (Sumner and Sutherland, 1973; Cull, 1974; Chen, 1978; de la Cruz et al., 1994; Johnson et al., 1998; Brännström and Kellerth, 1999). Loss of presynaptic inputs was also found in invertebrate motoneurons innervating the locust leg (Horridge and Burrows, 1974), suggesting a conserved mechanism. The same phenomenon was described in some central neurons in which transection axotomies are experimentally feasible: spinocerebellar neurons (Chen et al., 1977), abducens internuclear interneurons (Pastor et al., 2000), and Mauthner cells (Wood and Faber, 1986).

Most studies interpreted EM images according to the hypothesis that microglia is responsible for “stripping” synapses, however, in none of these ultrastructural studies it was possible to determine whether microglia actively removed the synapses or just occupied space vacated by lost synapses. Discrepancies in the interpretation of the role of microglia can be found in some of the contemporary studies. In the rat hypoglossal nucleus, two phases of glia coverage were distinguished: a first phase reactive to axotomy and characterized by microglia coverage (up to 2 weeks post-axotomy) and a second phase during motoneuron regeneration in which the cell is covered by astrocytes (Sumner and Sutherland, 1973). Other analyses in the same model emphasized astrocytic coverage from the start, noting relatively few microglia (Reisert et al., 1984). The latter was consistent with EM observations in cat spinal motoneurons, in which the microglia reaction is weaker compared to rodents (Cova and Aldskogius, 1984, 1985, 1986) enabling parsing out synapse detachment vs. microglia coverage (Chen, 1978). Axotomized sympathetic postganglionic neurons are also subject to synapse detachment but in this case, there is no microglia in the ganglia and detached synaptic boutons can be recognized at a distance from the cell body because, remarkably, the presynaptic active zone (PAZ) remains intact (Matthews and Nelson, 1975; Purves, 1975). Synaptic boutons detached from cat spinal motoneurons after sciatic nerve injuries disassemble their PAZs, but they are still recognizable in the vicinity of the motoneuron cell body frequently isolated by layers of astrocytic lamellae (Chen, 1978). Thus, alternative explanations for the loss of synapses included the involvement of astrocytes or cell-autonomous remodeling of postsynaptic membranes leading to synapse detachment (Sumner and Sutherland, 1973; Sumner, 1975a,b,c; Chen, 1978). The idea of cell-autonomous synaptic shedding lacked precise mechanistic explanations at the time, and the proposal that microglia (or astrocytes) physically remove synapses from the cell body prevailed and became the accepted hypothesis that continues to be cited in many past and present reviews (Kreutzberg, 1996; Moran and Graeber, 2004; Cullheim and Thams, 2007; Kettenmann et al., 2013; Spejo and Oliveira, 2015; Chen and Trapp, 2016).

## Microglia Is Not the Universal “Synaptic Stripper” of Adult Axotomized Motoneurons

Experiments to evaluate the microglia hypothesis were performed two decades after it was first proposed. The first test was a rather complex and indirect experiment that reduced microglia proliferation in the injured facial nucleus for a different goal: to reveal the microglia origins of brain macrophages (Graeber et al., 1989). In this study, the cytostatic agent adriamycin was injected in the facial nucleus, while at the same time the facial nerve received crush injuries combined with ricin application to induce motoneuron cell death and the appearance of brain macrophages. Adriamycin prevented microglia proliferation, the appearance of brain macrophages (therefore shown to derive from microglia in this model), and microglia migration to the surface of axotomized motoneurons. Qualitative EM observations of motoneurons treated with adriamycin revealed a normal complement of synapses after nerve crush. The authors concluded that synapse preservation in the absence of perineuronal microglia supported a role for microglia detaching synapses, but this conclusion was complicated by the effects of adriamycin on motoneuron metabolism and function (Bigotte and Olsson, 1983, 1984, 1987). Moreover, this observation was not supported in following experiments using quantitative approaches. One experiment blocked microglia proliferation and surface coverage of hypoglossal motoneurons by continuous infusion with mini-osmotic pumps of the anti-mitotic agent cytosine-arabinoside (ARA-C) and this resulted in no significant change in the number of synapses lost after axotomy (Svensson and Aldskogius, 1993). Another piece of evidence came from analysis of axotomized facial motoneurons in osteoporosis op/op mice that carry a spontaneous mutation disturbing expression of colony-stimulating factor 1 (CSF1; Kalla et al., 2001). These mice show decreased basal microglia numbers and after nerve injury, they display reduced microglia proliferation, lower expression of microglial activation markers and lack of coverage of the motoneuron surface (Raivich et al., 1994; Kalla et al., 2001). No differences in synaptic stripping were found between op/op mice and controls despite a blunted microglial response. Recent studies confirmed that CSF1 is upregulated in axotomized spinal motoneurons and is a necessary signal for microglia proliferation and migration towards the motoneuron surface (Akhter et al., 2019; Rotterman et al., 2019). Synaptic stripping proceeded normally in the absence of microglia interactions with the surfaces of axotomized motoneurons lacking *csf1*. In this experiment basal microglia numbers and nerve injury-induced activation of dorsal horn microglia were preserved, restricting the effects to microglia proliferation and activation in the ventral horn.

In conclusion, synaptic stripping was unaltered in several different experiments that prevented or modified microglia activation and their interaction with the cell bodies of hypoglossal, facial, or spinal motoneurons. Comparative analyses between species and mouse strains lead to similar conclusions. Transgenic mouse models with increased or decreased synaptic stripping over spinal motoneurons after sciatic injuries exhibited similar microglia reactions (Berg et al., 2013). Conversely, C57BL/6N mice display a higher microglia reaction around axotomized motoneurons compared to Wistar rats, despite lower synaptic stripping and functional loss (Yamada et al., 2008, 2011). The accumulated data confirmed long-held doubts about the microglia synaptic stripping hypothesis (reviewed in Aldskogius and Kozlova, 1998; Perry and O’Connor, 2010; Aldskogius, 2011) and suggest that the primary role of microglia around axotomized motoneurons is unlikely related to the induction of synapse stripping, although they probably exert modulatory roles as reviewed below.

## Role of Astrocytes in Synaptic Remodeling Around Axotomized Motoneurons

The EM observation of astrocytic processes covering the surface of motoneurons and enveloping synaptic boutons implicated them in synaptic stripping (summarized in Figure 1). Astrocytes around axotomized motoneurons do not proliferate but augment in size by increasing expression of glial fibrillary acid protein (GFAP) and vimentin (Sumner and Sutherland, 1973; Chen, 1978; Reisert et al., 1984; Graeber and Kreutzberg, 1986, 1988; Graeber et al., 1988; Tetzlaff et al., 1988; Gilmore et al., 1990; Svensson et al., 1994). Later, they extend sheet-like processes to cover the surface of axotomized motoneurons isolating their cell bodies from the rest of the neuropil and replacing microglia. Astrocytic lamellae also envelop detached synapses, but never engulf or degrade them. Synapse recovery following successful regeneration in the periphery coincides with the disappearance of astrocyte wrappings. If motoneurons are prevented from reinnervating muscle, the ensheathing of their cell bodies by astrocytes persists for long periods (Sumner, 1977a; Graeber and Kreutzberg, 1988; Laskawi and Wolff, 1996). Taking advantage of the astrocyte reaction around rat hypoglossal motoneurons being secondary and dependent on microglia activation, it was shown that altering astrogliosis did not affect synaptic stripping (Svensson et al., 1993). Similarly, synaptic stripping on spinal motoneurons was not prevented in dual GFAP and vimentin knockouts with reduced astrogliosis (Berg et al., 2013). However, in these animals around 35% more synapses were found on axotomized spinal motoneurons. Astrocytic reactions vary with mouse strain: A/J mice show stronger reactions around axotomized motoneurons compared to C57BL/6J mice and this is correlated with fewer synapses after axotomy and impaired recovery (Emirandetti et al., 2006). Mutant mice with variations in astrocytosis levels after nerve injury showed co-related variations in the amount of synaptic loss (Victorio et al., 2010; Freria et al., 2012; Ribeiro et al., 2019). A more definitive study for proving causality used transection of the facial nerve and blocked the astrocytic reaction by astrocyte-specific deletion of *STAT3* (Tyzack et al., 2014). This resulted in reduced GFAP upregulation, fewer astrocytic lamellae extensions and decreased motoneuron cell body coverage. Surprisingly it also caused a larger and more permanent loss of synapses due to reduced production of thrombospondin 1 (TSP-1). TSP-1 is a well-known regulator of *de novo* synaptogenesis during normal development and after pathology (reviewed in Eroglu and Barres, 2010). The diversity of reported effects on synaptic coverage after altering the astrocytic reaction around axotomized motoneurons could be explained considering two sequential roles for astrocytes. First, during the regenerative phase (when the axon is growing in the peripheral nerve) enlarged astrocytes isolate pre and postsynaptic surfaces preventing synapse re-formation, and also providing trophic support (Tyzack et al., 2014; Jones et al., 2015). Second, after motor axons reinnervate muscle, astrocytes withdraw their processes exposing motoneuron surfaces that then become available for synaptogenesis actively promoted through TSP-1. Therefore, although astrocytes are most likely not directly involved in the initial phase of synapse stripping, their activity influences synapse recovery in the regenerating motoneuron.

## Membrane Remodeling in Axotomized Motoneurons and Synaptic Stripping

The reviewed data suggest that neuron-glia interactions are not critical for the induction of synaptic stripping in axotomized motoneurons. EM support for the hypothesis of active postsynaptic membrane remodeling leading to synapse loss was suggested in early EM studies (see above) and later quantified over abducens motoneurons undergoing synaptic stripping induced by botulinum toxin (Pastor et al., 1997; Moreno-López et al., 1998). This model mimics synaptic changes occurring after the axotomy of motoneurons in the absence of injury and a microglia reaction (Sumner, 1977b). In this model, the first evidence of synapse detachment on the motoneuron cell body surface is the early separation of pre- and post-synaptic membranes in non-junctional areas. This is paralleled by a 3-fold increase in coated vesicles in the non-junctional postsynaptic membrane away from the postsynaptic density (PSD). Dissolution of inhibitory PSD gephyrin clusters and synaptic complexes occurs after much of the synaptic bouton has detached from the postsynaptic cell (Moreno-López et al., 1998). Finally, the motoneurons become covered by glial processes (Pastor et al., 1997; Moreno-López et al., 1998).

Overall, the EM observations suggest that synaptic stripping proceeds in three steps: (1) an increase in uptake of material from the membrane surface that correlates with reduced synaptic bouton adhesion throughout the non-junctional apposition; (2) dissolution of the PSD and PAZ and complete detachment of the synaptic bouton; and (3) coverage of pre and postsynaptic surfaces by glia (Figure 1). Work in the lab of Dr. Steffan Cullheim (Karolinska Institute) systematically cataloged in spinal motoneurons the expression of several synaptic adhesion (SynCAM 1–4, nectins 1 and 3, NCAM, N-cadherin, and Netrin-G2-ligand) and synaptic organizing molecules (PSD95, neuroligins 1–3) before and after sciatic nerve transection (Zelano et al., 2006, 2007, 2009a,b; Berg et al., 2010). This work generated a molecular picture that strikingly parallels the EM observations. A loss of synaptic adhesion in axotomized spinal motoneurons correlates with the early downregulation of mRNAs for SynCAM1, neuroligin-2 and -3 and Netrin-G2-ligand (Zelano et al., 2007; Berg et al., 2010). SynCAMs are involved, among other functions, in synaptic bouton adhesion through non-junctional sites (Kakunaga et al., 2005). Neuroligins, conversely, induce the formation of inhibitory and excitatory synaptic junctions and contribute to their functional and structural stability (Craig and Kang, 2007; Südhof, 2008). Netrin-G2-ligand interacts with PSD95 and regulates synapse number of specific subsets of excitatory synapses expressing netrin-G2 (Kim et al., 2006; Matsukawa et al., 2014). Synaptic adhesion is also modified by changes in the localization of adhesion proteins. N-cadherin mRNA expression was unaltered by axotomy, but the localization of the protein drastically changed from being clustered opposite to synaptic boutons on the cell bodies of intact motoneurons to be removed from this location after axotomy and shuttled to the regenerating axons (Zelano et al., 2006).

Proteins that organize PSD neurotransmitter receptor accumulations are downregulated with a slower time course compared to synaptic adhesion proteins. These include PSD95 at excitatory synapses (Che et al., 2000; Zelano et al., 2007) and gephyrin at inhibitory synapses (Moreno-López et al., 1998; Eleore et al., 2005b; Kim et al., 2018). The removal of these molecular organizers of excitatory and inhibitory synaptic PSDs is accompanied by changes in postsynaptic receptor expression that, as will be reviewed below, also occur with a time course slower than changes in synaptic adhesion. Altogether they induce dissolution of the PSD and synaptic complex after adhesion is reduced in non-junctional regions and thus fully detaching the synapse. These spaces are occupied by microglia first and astrocytes later (Figure 1).

Adhesion proteins of the nectin family have no basal expression in motoneurons but are quickly upregulated after axotomy (Zelano et al., 2006, 2009b). Upregulation of nectin-1 and -3 in Schwann cells and motoneurons, as well as nectin-like proteins 4 and 5 in motoneurons, could facilitate *cis* and *trans* interactions in the peripheral nerve during motor axon regeneration. Nectins are also expressed by astrocytes and are necessary for astrocytic support of neurons (Miyata et al., 2016). It is thus tempting to speculate that nectins and nectin-like proteins concurrently facilitate adhesion of the cell bodies and axons of regenerating motoneurons with respectively, astrocytes and Schwann cells. These possibilities should be fully investigated in the future.

Changes in adhesion proteins revert following muscle reinnervation and in coincidence with synapse restoration on the motoneuron cell body (Zelano et al., 2009a; Berg et al., 2010). In summary, bi-directional replacement of synapses and glia coverage over the membrane of motoneurons correlates with changes in expression and localization of cell adhesion proteins, a process that is coupled to regenerative mechanisms in the peripheral nerve. Nonetheless, other mechanisms might be at play since mouse models with increased or decreased synaptic stripping (MHCI KOs and C3 KOs, respectively) did not show modifications in adhesion protein plasticity after axotomy (Berg et al., 2013). Alternatively, reduced synaptic adhesion could be interpreted as permissive, but not sufficient for complete synapse retraction. Determining whether these changes are necessary will require specific manipulations of adhesion protein expression in axotomized motoneurons during synaptic stripping. Future studies will also need to fit the idea of global changes in synaptic adhesion with the different susceptibilities of inhibitory and excitatory synapses to detachment (see below) and the maintenance of synapses throughout most of the dendrite.

A further mechanism inducing detachment of synaptic boutons from postsynaptic membranes involves nitric oxide (NO) disruption of the actin cytoskeleton in presynaptic boutons causing their retraction from axotomized motoneurons (reviewed in Moreno-López et al., 2011). Neuronal nitric oxide synthase (nNOS) is upregulated in cranial, but not spinal motoneurons, after a variety of peripheral nerve injuries (Yu, 1994, 1997; Sunico et al., 2005; Liu et al., 2006). nNOS upregulation was shown to be necessary for synaptic stripping in the hypoglossal nucleus: blocking nNOS with the generalized NOS antagonist L-NAME, the specific nNOS inhibitor 7-nitroindazole, or abrogating nNOS upregulation by overexpressing miR-shRNA for nNOS with lentiviral vectors all blocked synaptic stripping on hypoglossal motoneurons after nerve crush (Sunico et al., 2005; Montero et al., 2010). Synaptic preservation affected only excitatory synapses, since inhibitory synapses are not removed from adult hypoglossal motoneurons after axotomy (Sumner, 1975a; Sunico et al., 2005). nNOS expression was also found sufficient for inducing synaptic stripping. AAV transduction of intact hypoglossal motoneurons with nNOS caused a loss of excitatory synapses in the adult, and interestingly, induced additional loss of inhibitory synapses in neonates (Sunico et al., 2010), suggesting developmental changes in susceptibility to stripping. NO generated by axotomized motoneurons acts in a spatially restricted paracrine manner on overlying synaptic boutons by stimulating guanylyl cyclase (GC), production of cGMP and activation of cGMP-dependent protein kinase (PKG). Thus, treatment with a membrane-impermeable NO scavenger (preventing paracrine action) or inhibitors of GC or PKG, preserved excitatory synapses (Sunico et al., 2005). PKG targets were identified as Rho kinase (ROCK) and its substrate myosin light chain (MLC). Phosphorylated MLC correlated with excitatory synapse withdrawal, and two specific ROCK inhibitors prevented this synaptic loss (Sunico et al., 2010). One result of p-MLC is actomyosin contraction and reorganization of the peripheral F-acting cytoskeleton (Svitkina et al., 1997), a mechanism associated with neurite retraction (reviewed in Newey et al., 2005). It was then proposed that actomyosin activation could induce synaptic bouton deformation and withdrawal explaining EM images showing bouton curvatures and separations in non-junctional areas (Moreno-López et al., 2011). Both isoforms of ROCK (ROCKα and ROCKβ) localized preferentially to excitatory synapses, pointing to a property that might confer differential susceptibility to stripping.

These studies made a compelling case for the actions of NO on the stability of excitatory inputs on hypoglossal motoneurons, however, it is not universally applicable. Spinal motoneurons undergo synaptic stripping after crush and transection of peripheral nerves without the upregulation of nNOS (Zhang et al., 1993; Yu, 1994). Spinal motoneurons upregulate NO only after ventral root avulsion (Wu et al., 1994a,b), a type of injury that induces enhanced synaptic stripping, affecting especially excitatory synapses (Linda et al., 2000; Novikov et al., 2000; Oliveira et al., 2004) and also motoneuron death (Koliatsos et al., 1994). The NO/GC/PKG/ROCK pathway for synapse detachment might thus operate in spinal motoneurons after very proximal nerve injuries and have an additive effect, inducing larger stripping of excitatory synapses.

## Differences in Synapse Removal and Maintenance According to the Type of Synapse and Motoneuron

The proportion of excitatory and inhibitory synapses removed from the cell body of different types of motoneurons after axotomy is variable. Only excitatory synapses are removed on hypoglossal motoneurons (Sumner, 1975a), in contrast, inhibitory synapses are strongly stripped from abducens motoneurons (Delgado-Garcia et al., 1988). In the spinal cord, gamma motoneurons lose more synapses than alpha motoneurons after the same injury and the loss of inhibitory synapses over gamma motoneurons is two-fold higher compared to excitatory synapses (Johnson and Sears, 1989). The loss of inhibitory and excitatory synapses is similar over cat medial motor column (MMC) thoracic spinal cord alpha motoneurons (Johnson and Sears, 1989), while inhibitory synapses are preferentially preserved on the cat and rodent lumbar lateral motor column (LMC) motoneurons in which excitatory synapse losses increase with injury proximity (Linda et al., 1992, 2000; Brännström and Kellerth, 1998; Novikov et al., 2000; Oliveira et al., 2004; Alvarez et al., 2011).

Synapse recovery also differs between excitatory and inhibitory inputs. In a comparative study of glutamatergic excitatory (VGLUT2) and GABA/glycine inhibitory (VGAT/VIAAT) synapses over medial gastrocnemius (MG) spinal motoneurons (a lumbar LMC pool) after tibial nerve transections in which regeneration was allowed (cut and repair) or not (cut and ligation), VGAT/VIAAT were lost to a lesser extent and recovered independently of muscle reinnervation (Alvarez et al., 2011). In contrast, VGLUT2 synapses were lost in larger numbers and recovered only after the motoneurons reinnervated muscles. This result suggests different requirements on peripheral factors for synapse detachment and recovery. The idea that synapse withdrawal after axotomy depends on trophic factors from peripheral targets has a long history. The earliest studies used silver methods to identify synaptic boutons around axotomized motoneurons and showed that blocking retrograde transport in peripheral nerves induced synapse withdrawal similar to axotomy, while functionally decoupling motoneurons from muscle (blocking impulse transmission in the nerve) did not (Cull, 1974). These findings were replicated on postganglionic sympathetic neurons whose axons were treated with colchicine (Purves, 1976). Target-dependence of motoneuron properties became a very active area of research with many comprehensive reviews on the topic (Mendell, 1984; Titmus and Faber, 1990; de la Cruz et al., 1996; Terenghi, 1999; Navarro et al., 2007; Benítez-Temiño et al., 2016). Here, we will focus on how these studies inform about differences in the removal and recovery of different synapses.

The two most thoroughly investigated neurotrophins concerning synaptic plasticity on axotomized motoneurons are Brain-Derived Neurotrophic Factor (BDNF) and Neurotrophin-3 (NT3). Continuous delivery of BDNF in the spinal subarachnoid space after ventral root avulsion did not prevent synaptic stripping evaluated with EM but facilitated synapse recovery in the absence of muscle reinnervation, especially inhibitory synapses (Novikov et al., 2000). NT3 applied to cut nerves preserved glutamatergic EPSPs on motoneurons from Ia afferent synapses (Mendell et al., 1999; more on this input later). NT3 and BDNF have now been proposed to mediate the effects of treadmill exercise on the preservation of synapses over axotomized spinal motoneurons (Krakowiak et al., 2015; Arbat-Plana et al., 2017).

A comprehensive direct comparison of the effects of different neurotrophins on preservation and recovery of specific synaptic inputs was carried out by the group of Dr. Angel Pastor (University of Seville) using as a model the axotomy of abducens motoneurons. This work draws on extensive knowledge about the synaptic inputs controlling tonic and phasic firing of abducens motoneurons in relation to eye position, eye velocity, and vestibular stimulation (reviewed in Benítez-Temiño et al., 2016). NT3 and BDNF were applied to the cut nerve at the time of injury (to test preservation) or after a 2-week delay allowing synaptic stripping (to test recovery). In all experiments, regeneration in the periphery was prevented, disallowing synapse recovery because of muscle reinnervation. NT3 and BDNF both preserved and recovered excitatory and inhibitory synapses in the absence of target reinnervation, but NT3 had preferential actions on phasic synaptic inputs modulating firing to eye velocity and BDNF on tonic inputs related to eye position (Davis-López de Carrizosa et al., 2009). Interestingly, excitatory vestibular inputs were recovered by BDNF and inhibitory vestibular inputs by NT3. In contrast to other motoneurons, abducens motoneurons are unique in that they also express TrkA (Morcuende et al., 2011). Nerve Growth Factor (NGF) activation of TrkA receptors (with p75^NTR^ blocked) recovered all synapses and synaptic modulation from all inputs, although synaptic gains were abnormally enhanced (Davis-López de Carrizosa et al., 2010). Recently, this same group showed that Vascular Endothelial Growth Factor (VEGF) also recovers all inputs and synapses on abducens motoneurons and results in firing modulation in injured motoneurons that is indistinguishable from control (Calvo et al., 2018). Conversely, astrocytic coverage of axotomized abducens motoneurons was reduced by BDNF, NT3, NGF and VEGF. Similarly, microglia around spinal cord motoneurons is reduced by BDNF (Novikov et al., 2000; Rodrigues Hell et al., 2009). These studies suggest that: (1) trophic factors applied to the motoneuron cell body or cut axon increase synapse preservation/recovery while reducing glia coverage; (2) trophic actions on synapse preservation/recovery can be redundant; and (3) some trophic factors display preferences for specific inputs, suggesting differences on mechanisms that remove or recover specific synapses.

## Immune System Signaling Mechanisms, Microglia and Synaptic Plasticity of Inhibitory Synapses

Searches for immune system genes involved in motoneuron cell death after axotomy revealed the upregulation of MHC-I expression in spinal and facial motoneurons after nerve injuries and with the independence of cell death (Maehlen et al., 1989). In particular, β2-microglobulin, a component of the MHC-I complex required for surface expression and signaling, is dramatically increased after axotomy (Linda et al., 1998). Later, an unbiased screen for genes regulated by synaptic activity in visual pathways during the formation and maintenance of ocular dominance columns identified MHC-I as a critical gene involved in synaptic plasticity (Corriveau et al., 1998; Shatz, 2009). This finding prompted analysis of β2 KO mice after sciatic nerve injuries and, unexpectedly, a larger loss of specifically inhibitory synapses was observed (Oliveira et al., 2004). While this implied a protective role of MHC-I activity on inhibitory synapses, subsequent studies found that enhancing MHC-I upregulation by combining interferon treatment with axotomy, also resulted in excessive loss of inhibitory synapses (Zanon and Oliveira, 2006). Thus, inhibitory synapse protection by MHC-I may require finely balanced levels of MHC-I and the effects also depend on the cell types and locations involved.

Surprisingly, analyses of MHC-I protein localization in axotomized motoneurons found no increases in the cell body or dendrites, instead, newly produced MHC-I was trafficked to the regenerating axons (Thams et al., 2009). Within the spinal cord, MHC-I protein was mainly found in activated microglia surrounding axotomized motoneurons. Microglia also express MHC-I receptors (reviewed in Thams et al., 2008) and intriguingly, β2 KOs showed an exaggerated microglial reaction the first few days after the injury that returned to normal levels within the first week (Cartarozzi et al., 2019). Microglia MHC-I is regulated by Toll-like receptor (TLR) activation. Global genetic deletion of TLR4 (KO) resulted in reductions of inhibitory synapse coverage of motoneurons cell body surfaces after axotomy while global deletion of TLR2 did the opposite; surface coverage by inhibitory synapses was larger in TLR2 KO axotomized motoneurons compared to wild-types. In both cases, there were reportedly no differences in bouton numbers being the changes in synaptic coverage explained by larger or smaller bouton sizes (Freria et al., 2012). These results need to be interpreted cautiously because inhibitory synapses also showed altered bouton sizes on uninjured motoneurons in these global KOs. Synapse bouton size is regulated during development and correlates with synaptic strength (Walmsley et al., 1998), thus size might influence synapse fates after injury in the adult. Nevertheless, a relationship between microglial activation and inhibitory synapse stability agrees with results suggesting that the rescue of inhibitory synapses on axotomized motoneurons by BDNF is paralleled by a decreased microglia reaction (Novikov et al., 2000; Rodrigues Hell et al., 2009). Similarly, studies in the cerebral cortex suggest that somatic wrapping of neurons by lipopolysaccharide (LPS)-activated microglia is neuroprotective through a mechanism that involves the removal of perisomatic GABA synapses and upregulation of anti-apoptotic genes (Chen et al., 2014). In contrast, inhibitory synapses are generally preserved over axotomized motoneurons and any neuroprotective role for microglia is at present controversial (reviewed in Aldskogius, 2011, but see Jones et al., 2015 and Tanaka et al., 2017). Regardless of the exact roles played by inhibitory synapses, MHC-I and TLRs have emerged as key modulators of inhibitory synapse plasticity after axotomy, potentially through microglia actions.

The last decade also highlighted the classical complement cascade through C1q and C3 as a canonical pathway for synapse opsonization and microglia-dependent removal of excitatory synapses during developmental pruning and in several pathologies (Stevens et al., 2007); reviewed in Stephan et al., 2012). C1q and C3 upregulation around hypoglossal, facial and spinal motoneurons are consistent findings after nerve injury (Svensson and Aldskogius, 1992; Svensson et al., 1995; Mattsson et al., 1998; Berg et al., 2012) although their cellular origins might differ. In all studies, C1q was found in microglia, but C3 was detected in microglia around injured brainstem motoneurons and astrocytes around injured spinal motoneurons. Synapse stripping on spinal motoneurons was altered in C3 KOs but not in C1q KOs, and the action was again biased towards inhibitory synapse preservation, while excitatory synapses (VGLUT2) were lost at normal levels (Berg et al., 2012). Thus, in contrast to complement-mediated microglia pruning of excitatory synapses in other brain regions during normal development or pathology, the removal of excitatory synapses from the cell body of adult axotomized motoneurons is independent of complement. This should not be too surprising since complement-mediated microglia removal of excitatory synapses occurs through engulfment and phagocytosis (reviewed in Stephan et al., 2012) and this is not the mechanism of synapse removal from motoneuron cell bodies after axotomy.

It should be emphasized that MHC-I and C3 effects occur over the subset of inhibitory synapses that undergo plasticity after axotomy. It is presently unknown if these correspond to specific types of inputs and how this relates to the diverse fates of inhibitory synapses on different types of axotomized motoneurons. Moreover, the known effects of C3 in peripheral nerves during regeneration (reviewed in Ramaglia et al., 2008) and of MHC-I on the neuromuscular junction (reviewed in Cullheim and Thams, 2010) cannot be dismissed when using global KOs. It is conceivable that actions in the periphery could influence the stability of inhibitory synapses centrally. In summary, mechanisms linking MHC-I, C3, and glia to inhibitory synapse plasticity deserve further study, but it is already established that these mechanisms differentiate between inhibitory and excitatory synapses on axotomized motoneurons.

## Functional Correlates and Significance of Differential Removal of Excitatory and Inhibitory Synapses

Differential removal of excitatory and inhibitory synapses predicts E/I imbalances that were hypothesized to promote motoneuron survival and/or the induction of a regenerative phenotype (Barron, 1989; Carlstedt and Cullheim, 2000; Navarro et al., 2007). The argument is that abolishing electrical activity focuses motoneuron resources on protein synthesis related to axon regeneration. Indeed, MHC-I and C3 KO mice with increased inhibitory synapse preservation correlate with faster axon regeneration and recovery of motor function (Oliveira et al., 2004; Berg et al., 2012); however, alternative interpretations due to possible effects in the periphery cannot be ruled out. MHC-I plays a critical role in neuromuscular junction development and stability (Thams et al., 2009; Cullheim and Thams, 2010), and many cellular elements of peripheral nerves regulate complement protein expression after injury (de Jonge et al., 2004). Complement inhibition has a variety of effects on peripheral regeneration, including acceleration of axon growth in certain situations (Ramaglia et al., 2008, 2009). Importantly, the role played by inhibitory synapses on regenerating motoneurons might be more complex than anticipated (see below). To better understand the outcomes of synapse stripping, it is important to consider first the functional changes associated with the loss of synapses in regenerating motoneurons.

The evidence for reduced excitatory synapse function on axotomized motoneurons is strong. Frequency and amplitude of spontaneous excitatory postsynaptic currents (sEPSCs) recorded in whole-cell mode *ex vivo* in brainstem slices initially increase at day 1 post-injury and then slowly decay below normal levels from 3 to 14 days postinjury in facial (Ikeda and Kato, 2005), vagal (Yamada et al., 2008), and hypoglossal rodent motoneurons (Yamada et al., 2011). The changes in frequency were large, while changes in amplitude were smaller and sometimes failed to reach significance. No changes were detected in the size of quantal events (miniature (m)EPSCs recorded in tetrodotoxin). These results indicate a decrease in the number of presynaptic release sites or release probability without a large alteration in postsynaptic sensitivity to retained synapses. This matches the anatomical loss of synapses but is not consistent with the downregulation of postsynaptic AMPA, NMDA, and metabotropic glutamate receptors regularly reported in many different types of axotomized motoneurons (Piehl et al., 1995; Popratiloff et al., 1996; Alvarez et al., 1997, 2000; Kennis and Holstege, 1997; Tang and Sim, 1997; García Del Caño et al., 2000; Nagano et al., 2003; Eleore et al., 2005a). Reduced glutamate receptor expression is significant in rat motoneurons 7 days after axotomy coinciding with the dissolution of PSDs from excitatory synapses on the cell body. This downregulation is, however, subunit-specific, being intense for GluA2 and GluA3 but mild for GluA4, suggesting the appearance of calcium-permeable AMPA receptors in adult motoneurons (Alvarez et al., 2000; Eleore et al., 2005a). It is thus possible that while the synaptic current amplitude is not much changed in remaining synapses, their properties are significantly altered. Remarkably, AMPA receptor subunit expression returns to normal in facial, hypoglossal and spinal motoneurons 45–60 days post-axotomy even when muscle reinnervation does not occur (Kennis and Holstege, 1997; García Del Caño et al., 2000; Eleore et al., 2005a). Thus, recovery of normal AMPA receptor subunit expression is not necessarily correlated with synapse recovery, but might influence the function of retained synapses resistant to stripping. Interestingly, the time-course of AMPA subunit regulation parallels changes in regenerative capacity in rat spinal motoneurons (Fu and Gordon, 1995). This coincidence might reflect switches in the genetic program for regeneration or, perhaps more enthrallingly, a causal relationship between AMPA subunit expression and motor axon regeneration.

Changes in synaptic function recorded *ex vivo* in slices correlate with *in vivo* alterations in firing modulation of spinal and brainstem motoneurons by excitatory inputs after axotomy. Hypoglossal motoneurons show decreased firing modulation in response to excitatory inspiratory drive and hypercapnia (Sunico et al., 2005), while abducens motoneurons show decreased synaptic drive regulating firing according to eye velocity and position and vestibular input (Delgado-Garcia et al., 1988; Davis-López de Carrizosa et al., 2009, 2010). Analyses of excitatory inputs over axotomized spinal motoneurons overwhelmingly centered on analyses of the synapse between muscle-stretch sensitive Ia afferents and motoneurons in the anesthetized cat spinal cord. As will be reviewed later, the behavior of Ia-motoneuron synapses is a special case because, frequently, the presynaptic Ia sensory afferent is co-injured with the motor axon in the peripheral nerve. In situations in which the postsynaptic motoneuron is axotomized and the presynaptic Ia afferent axon left intact, the compound Ia monosynaptic EPSP showed an increase in amplitude during the first 3 days post-axotomy (Miyata and Yasuda, 1988; Seburn and Cope, 1998; Bichler et al., 2007) followed by a period in which rise time and amplitudes of the Ia EPSP gradually decreased (Eccles et al., 1958; Kuno and Llinas, 1970) and connectivity between Ia afferents and motoneurons significantly decreased (Mendell et al., 1976; see below).

In summary, there are complex changes in glutamatergic synaptic function during the first few days after axotomy that are followed by a consistent depression that correlates with the physical removal of synapses from the cell body and proximal dendrites. This should not be interpreted as a major anatomical loss of excitatory synapses, since >90% of the total input to motoneurons is distributed throughout dendrites (Rose and Neuber-Hess, 1991; Brännström, 1993; Starr and Wolpaw, 1994; Bae et al., 1999). To be sure, the dendritic arbor retracts and this occurs at the expense of distal and intermediate dendritic regions causing a 30% decrease in the available membrane at locations were excitatory synapses predominate (Sumner and Watson, 1971; Brännström et al., 1992; Brännström and Kellerth, 1998). Thus, while there is some loss of mid and distal dendritic synapses, most are retained and synaptic densities on dendrites do not change much (Delgado-Garcia et al., 1988; Brännström and Kellerth, 1998). After axotomy motoneurons also show increased excitability with decreased rheobase, increase input resistance and sometimes lower firing thresholds (reviewed in Mendell, 1984; Vanden Noven and Pinter, 1989; Titmus and Faber, 1990; González-Forero and Moreno-López, 2014). The lack of functional compensation by the synaptic drive on dendrites in these conditions is unexplained. Dendritic excitatory inputs could be affected by global changes in neurotransmitter receptor expression. Alternatively, dendritic synaptic integration mechanisms could be affected. It is well-accepted that propagation of EPSPs in the large dendritic arbors of motoneurons requires voltage-gated conductances to boost depolarizing currents and prevent their electrotonic decay before reaching the cell body (reviewed in Heckmann et al., 2005). Whether this mechanism is lost after nerve injury is unknown. Independent of mechanism, axotomy results in a period of reduced drive from glutamatergic synapses that coincides with the time the motoneuron regenerates or is attempting to regenerate its axon in the peripheral nerve. This functional depression reverts after motoneurons reinnervate muscle, in agreement with the hypothetical necessity of decreased excitatory activity on regenerating motoneurons. However, this does not mean that the motoneuron is electrically silent; GABA/glycine synapses can introduce a new source of excitatory drive, instead of further reducing it as has been assumed for decades.

**Figure 2:**
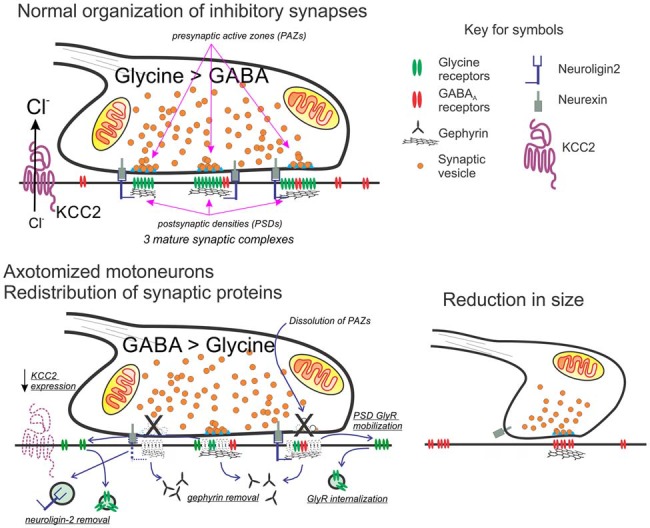
Working model for the molecular and structural reorganization of inhibitory synapses on the cell surface of axotomized motoneurons. Top: normal organization of mixed GABA/glycine synapses on adult motoneurons. Glycine content is higher than GABA in mature synapses on the cell body of motoneurons. Each synaptic bouton forms multiple synaptic complexes (usually >6) and each has a postsynaptic density (PSD) formed by a gephyrin scaffold that co-clusters GABA_A_ and glycine receptors, with glycine receptors at higher density in mature synapses. Neuroligin-2 interacts with gephyrin and binds presynaptic neurexin located adjacent to the release site or presynaptic active zone (PAZ). The direction of the ionic synaptic current is defined by the chloride concentration gradient maintained low intracellularly by the activity of KCC2. Bottom: after axotomy, neuroligin, gephyrin, and KCC2 are removed. Glycine receptors increase their mobility and internalization is unmatched by new membrane insertion since glycine receptor expression strongly decreases. In contrast, the expression of GABA_A_ receptors with α2 and β2/3 subunits is maintained. In the presynaptic bouton, there is an increase in GABA synthesis. Bouton size is also reduced and contains fewer synaptic complexes and these now operate with a significant GABAergic component (see references in the text).

The retention of inhibitory synapses on cell bodies of many types of motoneurons after axotomy, has been argued to promote inhibition. However, the function of GABA/glycine synapses depends not only on synaptic bouton numbers but also on the relative ratios of GABA and glycine release, the proportions of postsynaptic GABA_A_ and glycine receptors, the subunit composition of GABA_A_ receptors, the number of independent release sites and the driving forces through GABA_A_ and glycine receptors set by the internal chloride concentration (reviewed in Alvarez, 2017). Retained inhibitory synapses undergo profound changes (summarized in Figure 2) that are reflected in large reductions in the frequency of spontaneous inhibitory postsynaptic currents (sIPSCs), as described over mouse and rat hypoglossal and vagal motoneurons recorded *ex vivo* in a whole-cell mode in slices after axotomy (Yamada et al., 2008, 2011). This functional reduction occurs even though no reduction in inhibitory synapse coverage was detected in these motoneurons in EM or immunocytochemical studies. The results agree with data *in vivo* showing lower amplitudes of IPSPs evoked in hypoglossal and trigeminal motoneurons in the anesthetized cat by stimulation of the lingual nerve or cortex (Takata, 1981; Takata and Nagahama, 1983, 1984, 1986), as well as a reduction in inhibition of spinal motoneurons after stimulation of the antagonistic muscle nerve (Kuno and Llinas, 1970). Curiously, recordings performed after axotomy in hypoglossal motoneurons found suppression of IPSPs in many motoneurons and their replacement by EPSPs (Takata and Nagahama, 1983). This was interpreted at the time as higher dysfunction in inhibitory compared to excitatory synapses, despite anatomical evidence to the contrary. Nowadays, these results can be re-interpreted considering the downregulation of the potassium chloride transporter 2 (KCC2) in axotomized motoneurons (vagal: Nabekura et al., 2002; facial: Toyoda et al., 2003; Kim et al., 2018; hypoglossal: Tatetsu et al., 2012). Similar KCC2 loss occurs throughout cell bodies and dendritic arbors of spinal motoneurons and restoration depends on signals reporting muscle innervation (Akhter et al., 2019). Thus, a potential role of KCC2 loss on motor axon regeneration was proposed. KCC2 removal causes a depolarization shift of +19 mV in E_GABA_ in facial motoneurons (Toyoda et al., 2003) and +13.4 mV in vagal motoneurons (Nabekura et al., 2002), a difference that is probably explained by the lower basal expression of KCC2 in vagal motoneurons (Ueno et al., 2002). Analyses of facial motoneurons *ex vivo* (slices) demonstrated that this depolarization shift induces the reversal of GABA/glycine synaptic actions and appearance of low frequency spontaneous depolarizing GABA-mediated oscillations that activate NMDA and voltage-gated Ca^2+^ channels (Toyoda et al., 2003). The significance of these rhythmic GABA/NMDA/voltage-gated Ca^2+^ subthreshold calcium oscillations in adult motoneurons is currently unknown, but it is tempting to speculate that they might be part of the regeneration program. Similar rhythmic depolarizations are critical for many developmental processes including neurite growth (Ben-Ari, 2014) and are characteristic of early developing motoneurons (O’Donovan et al., 1998; Hanson et al., 2008; Czarnecki et al., 2014). Regenerating motoneurons might therefore not be electrically silent. On the contrary, they might replace high frequency glutamatergic excitatory synaptic depolarizations by low-frequency voltage oscillations that efficiently permeate calcium transients best adapted for promoting regeneration. The need for activity for optimal regeneration also agrees better with work that shows that electrical activity and exercise promote regeneration onset and axon growth speed (Gordon and English, 2016).

Axotomized motoneurons simultaneously show “inhibitory” synaptic bouton retention and lower frequency of spontaneous IPSCs, implying that release probability from retained synaptic boutons must be reduced. Individual “inhibitory” synapses on spinal and brainstem motoneuron cell bodies display multiple independent synaptic complexes, each one with an independent PAZ release site opposed by a gephyrin PSD cluster (Alvarez et al., 1997; González-Forero et al., 2004). Within this structural arrangement, a reduction in release probability likely occurs because of decreasing the number of release sites per bouton. Also, changes in GABA_A_ and glycine receptor subunit expression have been reported in facial motoneurons after axotomy (Eleore et al., 2005b; Vassias et al., 2005). mRNAs for α1, β2 and γ2 GABA_A_ subunits were decreased starting at day 3 post axotomy (although protein removal from the PSD lagged likely due to differences in mRNA and protein turnover). Changes in these subunits persisted through the last day of the study (60 days after injury) despite 80% re-innervation of muscle at this time point. In contrast, expression of GABA_A_ α2, β1, and β3 subunits was unchanged. Glycine receptor α1 and β subunits were also downregulated, but this was reversed with muscle re-innervation 60 days after injury. These results suggest the disappearance of fast glycinergic currents (and the fast α1 GABA subunit) during regeneration in favor of slower GABAergic synaptic phenotypes. Also, after a transient initial decrease in presynaptic GABA synthetic machinery, these changes are overturned (Kikuchi et al., 2018) and even increased, resulting in larger than normal GABA presynaptic content (Vaughan, 1994). These changes have not been generalized to other motoneurons, but raise the possibility that injury may induce the reversal of the normal maturation of mixed GABA/glycine co-releasing synapses from early slow GABAergic mechanisms better tuned for developmental processes to later faster glycinergic mechanisms adapted to signal processing (Gao and Ziskind-Conhaim, 1995; Singer and Berger, 2000; Russier et al., 2002). Accordingly, the most apparent change of IPSPs on axotomized spinal motoneurons *in vivo* is an almost doubling of their duration (Kuno and Llinas, 1970).

Changes in inhibitory and excitatory synapses on the cell body of axotomized motoneurons might thus relate to the development of a regenerative phenotype requiring slow depolarizing GABAergic activity while simultaneously lowering fast glutamatergic and glycinergic activity and perhaps also blocking synaptic actions on dendrites from reaching the cell body. Microglia might exert modulatory actions on inhibitory synapse numbers as reviewed above, and additionally promote this functional switch. For example, LPS-activated microglia promotes a glycine to GABA conversion of synapses on spinal neurons by increasing glycine receptor membrane mobility and reducing its anchoring to the PSD, while maintaining GABA_A_receptors. These changes are reflected in reduced glycinergic components in IPSCs with no change in the GABA_A_ component (Cantaut-Belarif et al., 2017).

## Proprioceptive Inputs Axotomized in the Peripheral Nerve Undergo Synaptic Plasticity That Is Permanent and Differ From Synapse Stripping

One key input to spinal motoneurons arises from Ia proprioceptive afferents and, to a lesser extent, group II afferents, informing about muscle length and dynamics and respectively innervating primary and secondary endings of muscle spindles. In all species and spinal cord regions examined, Ia afferents project segmentally into medial lamina V/VI (or Clarke’s Column in thoracic regions), lamina VII, and IX, where they make synapses on different kinds of interneurons and motoneurons (Brown and Fyffe, 1978; Burke et al., 1979; Ishizuka et al., 1979; Burke and Glenn, 1996; Nakayama et al., 1998; Vincent et al., 2017). In lamina IX, Ia afferents form dense synaptic arbors that make functional contacts with most motoneurons (>90%) in the homonymous motor pool (i.e., projecting to the same muscle; cat: Mendell and Henneman, 1968; rodent: Bullinger et al., 2011). They also make synapses with motoneurons innervating muscle synergists (similar motor action), though in this case the strength of the Ia EPSP and the connectivity index are both reduced (Scott and Mendell, 1976). The monosynaptic Ia-motoneuron connection forms the basis of the fast stretch-reflex and has been amply studied because its accessibility and because the reflex is easily tested and in the clinic is diagnostic of many neurological disorders characterized by hypo- or hyperreflexia.

Not surprisingly, Ia-motoneuron synapses were intensely investigated in the earliest electrophysiological studies of motoneurons after axotomy (Eccles et al., 1958, 1959; McIntyre et al., 1959; Kuno and Llinas, 1970; Mendell et al., 1974, 1976). The main observation was a reduction of the Ia EPSP amplitude and a slowing of its rise time and time-to-peak accompanied by an increase in duration estimated by their half-widths. Similar changes were observed both in response to nerve volleys synchronously activating many Ia fibers and in response to input from single Ia fibers. The changes were consistently observed when Ia afferents were axotomized by the nerve injury, independent of whether the postsynaptic motoneuron was axotomized or not, or the distance between axotomy and the sensory afferent cell body (Eccles and McIntyre, 1953; Eccles et al., 1959; Gallego et al., 1979, 1980; Goldring et al., 1980). Specific axotomy of motoneurons (sparing presynaptic Ia afferents) resulted in more variable results. Changes in Ia EPSPs amplitude and time course were profound and rapid when the injury was proximal to the cell body, like after ventral root section (Eccles et al., 1958; Kuno and Llinas, 1970), but had a lesser and more protracted effect if the motor axon was transected at a distal location, typically close to the muscle (Eccles et al., 1959; Mendell et al., 1974, 1976; Gallego et al., 1979, 1980; Goldring et al., 1980). Changes in Ia EPSP properties were first interpreted according to the expected decay in electrical signals in passive dendrites predicted by Rall’s cable-theory (Rall et al., 1967); decreased amplitudes along with slower rise times and increased half-widths of single Ia fiber EPSPs were explained as a change in the position of the synaptic input after axotomy from close to the cell body to more distal locations (Kuno and Llinas, 1970; Mendell et al., 1974). This was suggestive of the synapse stripping phenomenon. Changes in Ia EPSP properties preceded full disconnection of single Ia afferents from motoneurons (estimated by decreased percentages of motoneurons in the homonymous pool contacted by individual Ia fibers) suggesting that single Ia axons remove their proximal synapses before distal synapses and before total disconnection (Mendell et al., 1976). Similarities between Ia synapse plasticity and synaptic stripping was further supported by the recovery of Ia EPSP amplitudes after muscle re-innervation (Mendell and Scott, 1975; Gallego et al., 1980; Mendell et al., 1995).

Interpretation of the modifications of the Ia input as a case of synaptic stripping was proposed in the earliest study by Blinzinger and Kreutzberg (1968) in the facial nucleus and this view has continued unabated in reviews and commentaries to this day. However, there are important problems with this parallelism: (1) except trigeminal motoneurons (Yoshida et al., 1999), Ia afferent inputs do not exist on brainstem motoneurons, including facial motoneurons where this comparison was first made; (2) anatomically, only 1% of the synaptic coverage of motoneurons on the cell body corresponds to synapses of dorsal root origin (Conradi, 1969). Therefore, the fate of such a small proportion of synapses in EM studies was impossible to accurately predict without any means for identification; (3) the bulk of Ia synapses target dendrites (Burke et al., 1979; Brown and Fyffe, 1981; Redman and Walmsley, 1983a,b; Burke and Glenn, 1996), where they would be protected from synaptic stripping mechanisms as reviewed above; and (4) Ia EPSPs are similarly altered by damage of the presynaptic Ia afferent in the absence of motoneuron axotomy (Gallego et al., 1979).

Interpretation of changes in Ia EPSP amplitude and time course solely in terms of synapse location is more complex than initially predicted (Gustafsson and Pinter, 1984; Vanden Noven and Pinter, 1989). Moreover, direct comparisons of single Ia EPSPs with the dendritic locations of the synaptic boutons anatomically mapped on the reconstructed dendritic arbors revealed that the “unitary” Ia EPSP (from a single Ia bouton) amplitude and time course recorded at the soma is independent of dendritic location (Redman and Walmsley, 1983b). This was argued to occur because Ia synaptic conductances (postsynaptic sensitivities afforded by the number of glutamate receptor channels clustered in the PSD) were proposed to increase with distance from the cell body (Iansek and Redman, 1973; Jack et al., 1981). Another issue is that most early *in vivo* studies of Ia EPSP properties on axotomized motoneurons were done under anesthesia regimens that were later found to suppress synaptic integrative mechanisms of dendrites. These consist of voltage-gated persistent inward currents (PICs) that “boost” the amplitude of dendritic Ia synaptic currents close to four times, such that they effectively modulate firing at the initial segment (Lee and Heckman, 2000). PICs depend on neuromodulatory input arising from the brainstem which is suppressed by many forms of anesthesia (reviewed in Heckman et al., 2008). The status of Ia synapse PIC amplification after axotomy is unknown and its impact on EPSP amplitude and time course in axotomized and regenerating motoneurons needs further study.

A major problem was the lack of anatomical analyses of Ia synapses after nerve injuries. These did not occur until recently (Alvarez et al., 2010, 2011; Rotterman et al., 2014; Schultz et al., 2017) facilitated by the discovery of VGLUT1 as a marker of proprioceptive synapses in the ventral horn (Todd et al., 2003; Alvarez et al., 2004). VGLUT1 synapses in lamina IX and on motoneurons, are mostly Ia synapses, but a minority might also originate from type II afferents (Alvarez et al., 2011; Vincent et al., 2017). The synapses of Ib afferents innervating Golgi tendon organs and informing about muscle force are also VGLUT1 positive, but Ib afferents do not project to the ventral horn in cats or rodents (Brown and Fyffe, 1979; Vincent et al., 2017). Experiments in which both muscle afferents and motoneurons were simultaneously axotomized after tibial nerve injuries showed that the behavior of VGLUT1(Ia/II) synapses on motoneurons exhibited many differences with synaptic stripping and also with some of the conclusions derived from electrophysiological analyses of the Ia EPSP: VGLUT1(Ia/II) synapses are lost and reorganized throughout the whole dendritic arbor, not only the cell body, and the changes are not recoverable after the motoneuron reinnervates muscle (Alvarez et al., 2011; Rotterman et al., 2014). VGLUT1(Ia/II) synapses on rat motoneurons are normally distributed at high density in proximal and mid-distance dendrites (up to 400 μm from the cell body) with many forming tight clusters of closely grouped synapses. VGLUT1(Ia/II) synapses on distal dendrites occur at low density and in isolation from each other (Rotterman et al., 2014). Proximal VGLUT1(Ia/II) synapse clusters resemble earlier accounts of single Ia afferent axon terminal collaterals frequently establishing 2–5 closely spaced *en passant* or terminal synaptic boutons on cat motoneuron dendrites (Brown and Fyffe, 1981; Redman and Walmsley, 1983a,b; Burke and Glenn, 1996). Parallel physiological analyses led to the conclusion that these synaptic groupings increase the strength of dendritic Ia EPSPs (Redman and Walmsley, 1983a,b). VGLUT1(Ia/II) synapse loss provokes the disappearance of synaptic clusters and the resulting synaptic organization is one of individual, isolated, VGLUT1(Ia/II) synapses occurring at low-density throughout the dendritic arbor (Rotterman et al., 2014). This predicts a non-recoverable weakening of Ia synapses and likely disconnection of individual Ia afferents from a large proportion of motoneurons.

Lack of recovery of VGLUT1(Ia/II) synapses after nerve transection and regeneration was in apparent contradiction to the general assumption that Ia EPSPs recover after muscle re-innervation. This conclusion was first derived from analyses of injuries in 5–8 days old kittens (Mendell and Scott, 1975). We now know that the first week after birth is a critical period for the developmental maturation of this input (Mentis et al., 2006; Siembab et al., 2010; Vukojicic et al., 2019) introducing significant interpretation confounds. When Ia EPSP recovery was studied in adult cats the results depend on the type of injury-inducing axotomy and the postsynaptic motoneuron tested. Ia EPSP amplitude recovery in homonymous and heteronymous connections was complete (and even above normal values) when axotomy was induced by crushing the nerve close to the muscle (Gallego et al., 1980). After nerve transection at the same location EPSP amplitudes from injured and regenerated Ia afferents onto heteronymous uninjured motoneurons recovered to around 50% of their original size (Mendell et al., 1995) while homonymous Ia EPSPs recovered much less in injury models affecting both Ia afferents and motoneurons (Eccles et al., 1959). These results agree with the good recovery of VGLUT1(Ia/II) synapses after nerve crush in rats (Schultz et al., 2017) compared to the lack of recovery when transecting the same nerve (Alvarez et al., 2011). Interestingly, recovered VGLUT1 (Ia/II) synapses following nerve crush displayed reduced presynaptic GABAergic P-boutons (Schultz et al., 2017), suggesting decreased presynaptic inhibition. In rats, the presence after regeneration of compound electrically-evoked Ia EPSPs and their normal amplitude modulation during high-frequency firing was confirmed in several studies (Haftel et al., 2005; Alvarez et al., 2011; Bullinger et al., 2011), but their exact level of recovery remained unknown because these studies were not designed to measure maximal amplitudes. Partial recovery of the compound Ia EPSP amplitude implies strengthening of remaining synapses but without physical recovery of lost synapses: single Ia afferents remain functionally disconnected from many motoneurons and VGLUT1 synapses remain depleted on motoneuron dendrites (Bullinger et al., 2011; Rotterman et al., 2014).

The loss of VGLUT1(Ia/II) synapses on motoneurons are the result of the removal of their axons from the ventral horn, not merely detachment of synapses from the membrane of axotomized motoneurons. Intra-axonal fills with neurobiotin of single afferents that regenerated and recovered muscle stretch responses typical of Ia afferents demonstrated that their central projections did not reach lamina IX. They also displayed fewer collaterals than normal in lamina VII, while their density appeared normal in lamina V and VI (Alvarez et al., 2011; Bullinger et al., 2011; Rotterman et al., 2014). Ia axon retraction towards the dorsal horn causes denervation of the motoneuron cell body and dendrites in lamina IX and VII and might prevent Ia synapse recovery after muscle re-innervation. An alternative explanation to axon retraction is that muscle spindles become reinnervated by Ib axons instead of the original Ia afferents. Ib afferents are capable of innervating vacated muscle spindles and in doing so, their responses to muscle stretch become indistinguishable from the original Ia afferents (Banks and Barker, 1989), however, Ib afferents do not project to the ventral horn. One electrophysiological study in the cat concluded that muscle spindles are reinnervated after axotomy by a random mix of Ia and Ib axons (Collins et al., 1986). In this study, Ia afferents were defined as muscle afferents with axons in the ventral horn and capable of generating field potentials in lamina IX, while Ib afferents were defined as lacking this projection and capacity. Using these criteria, 89% of afferents responding to muscle stretch were found to project to the ventral horn in normal cats, while this percentage decreased to 53% at 3 months, 50% at 6 months and 41% at 9 months after injury and repair of the cat MG nerve (Collins et al., 1986). The level of muscle re-innervation by motor axons in this model is moderate around 3 months and almost complete 9 months after injury (Foehring et al., 1986). Afferents without ventral horn projections were interpreted as Ib afferents, but many could be Ia afferents that retracted their ventral horn projections. This interpretation also fits better with the progressive loss of stretch-sensitive afferents with ventral projections, continuing even after muscle reinnervation. Regardless of the mechanism, the results suggest a dramatic drop in connectivity between Ia afferents and motoneurons in the homonymous motor pool. Similar to cats, single Ia afferents in the rat establish monosynaptic connections with >90% of motoneurons in the pool, but 6 months to 1 year after injury of the MG nerve and regeneration this percentage is only 17% (Bullinger et al., 2011). Partial transection of the MG nerve in the cat also showed a drop to 50–70% connectivity between intact Ia afferents coursing in the spared nerve region with axotomized MG motoneurons 60–77 days after injury (Mendell et al., 1974, 1976). Differences between these studies stem from the fact that Ia afferent disconnection might be more profound when both Ia afferents and motoneurons are axotomized. Also, the data in the cat study was gathered at an earlier time point and thus might not have progressed to the same level of disconnection as in the rat study.

**Figure 3:**
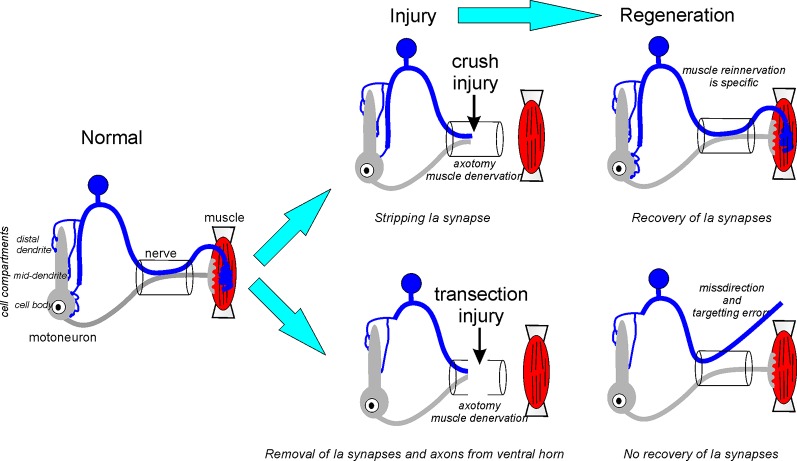
Fate of the Ia afferents synapses after crush or transection neve injuries. After nerve crush, the continuity of endoneurial tubes is preserved and guides sensory and motor axons during regeneration to the original targets. Centrally, Ia afferent synapses undergo normal synaptic stripping after nerve crush and then recover in coincidence with axon regeneration and muscle reinnervation in the periphery. After nerve transection, continuity is lost and the sensory or motor axon (or both) can be routed to the wrong muscles. Also, Ia sensory axons targeting the correct muscle can be misguided to the wrong muscle sensory receptor and/or fail to reinnervate any muscle spindle. After nerve transection, Ia axon terminal collaterals are removed from the ventral horn causing massive and permanent denervation of motoneuron cell bodies and proximal and mid-distance dendrites where the majority of Ia synapses reside. These synapses are not recovered after muscle reinnervation resulting in an enduring loss of connectivity between Ia afferents and motoneurons.

In conclusion, nerve injury affects both Ia afferents and motoneurons, and we believe the diversity of reported functional changes in the Ia-motoneuron synapse fits reasonably well within a dual mechanism model (summarized in Figure 3). First, motoneuron axotomy causes Ia synapse stripping along with other excitatory synapses that are more intense when lesions are proximal to the cell body. Also, axotomy of the Ia afferent triggers a second mechanism that induces the removal of Ia axon collaterals from the ventral horn. This process has a slower time course and results in the loss of Ia synapses also from dendrites, non-injured heteronymous motoneurons and likely other neurons in the ventral horn. This second mechanism is not reversible by muscle reinnervation and causes a permanent change in ventral horn circuitry. Ia axon die-back is dependent on the type of injury and does not occur after crush injuries. In this case, initial Ia synaptic stripping after motoneuron axotomy is reversible (Figure 3).

## Mechanisms of Ia Axon Removal From the Ventral Horn and Microglia Involvement

Genetic targeting of microglia activation after nerve injury demonstrated the necessity of specifically ventral horn microglia for permanent removal of VGLUT1(Ia/II) synapses from motoneuron cell bodies and dendrites (Rotterman et al., 2019). Abrogation of this microglia reaction rescues VGLUT1(Ia/II) synapses in regenerated motoneurons but does not prevent early transient stripping of these synapses along other excitatory synapses (labeled with VGLUT2) on the cell body. Thus, after transection nerve injuries, VGLUT1(Ia/II) synapses on the cell body of axotomized motoneurons undergo microglia-independent synapse stripping after which a different microglia-dependent mechanism induces the permanent loss of this input from dendrites.

Microglia actions are not a response to a degenerative mechanism intrinsic to the injured Ia afferent. Degeneration of central synapses and axons of trigeminal and spinal cutaneous sensory afferents axotomized by nerve injuries was detected in studies using silver-staining and/or EM to identify degenerating axons (Grant and Arvidsson, 1975; Knyihar and Csillik, 1976; Grant and Ygge, 1981; Aldskogius et al., 1985; Arvidsson et al., 1986). Some EM images even show possible microglia phagocytosis of these synapses (Arvidsson et al., 1986). The process was named “transganglionic anterograde degeneration.” In some of these studies, the disintegration of central synapses was related to axotomy-induced degeneration or even cell death of certain types of sensory neurons in the dorsal root ganglion after nerve injuries. While some small afferent neurons are indeed susceptible to degeneration and cell death, the larger skin mechanoreceptors and muscle proprioceptors are not (Arvidsson et al., 1986; Tandrup et al., 2000). Accordingly, after sciatic nerve injury, most degenerating axons are concentrated in lamina III with little evidence of degeneration-associated axon argyrophilia in the projection areas of proprioceptors in the deep dorsal horn (laminae V and VI) or ventral horn (Arvidsson et al., 1986). Thus, the disappearance of ventrally directed Ia axons is unlikely to be due to degenerative processes in the axon, at least of the type associated with increased argyrophilia.

After peripheral nerve injury, microglia activation occurs in all spinal cord and brainstem areas receiving central projections from injured afferents; for sciatic nerve injuries, these include the dorsal horn, Clarke’s column, and dorsal column nuclei (Eriksson et al., 1993). Some microglia activation might be associated with transganglionic degeneration of specific sensory afferents, but superficial laminae microglia activation in the spinal cord is also related to CSF1 release from sensory afferents terminating in this region (Guan et al., 2016). Ventral horn microglia activation depends only on CSF1 released by injured motoneurons, while signals from injured proprioceptors might be minimal (Rotterman et al., 2019). The relation of dorsal and ventral horn microglia with the central projections of peripherally injured, non-degenerating large afferents, differs. Anatomical remodeling of the ventral projections of neurobiotin-filled Ia axons (Alvarez et al., 2011) is not paralleled by similar removal of dorsal horn projections of neurobiotin-filled cutaneous mechanoreceptors after nerve injury and regeneration (Koerber et al., 2006). This also agrees with the maintenance of dorsal horn projections from injured proprioceptors. Dorsal and ventral microglia may interact differently with central axons of sensory afferents injured in the peripheral nerve and emerging data suggest that many properties of microglia after nerve injury differ between dorsal and ventral horns (Akhter et al., 2019; Rotterman et al., 2019).

The mechanism by which activated ventral microglia recognizes the spinal projections of Ia afferents injured in the periphery is unknown. During normal development, microglia-dependent synaptic pruning of excess VGLUT1(Ia/II) synapses occurs at specific postnatal critical periods through C1q opsonization (Vukojicic et al., 2019). C1q targets ineffective or silent synapses, and an exaggeration of this mechanism was proposed to be responsible for the loss of Ia afferent inputs on motoneurons in a mouse model of spinal muscular atrophy (Mentis et al., 2011; Vukojicic et al., 2019). C1q removal did not alter synapse stripping after nerve injury in adult mice (Berg et al., 2012), but this study did not analyze VGLUT1(Ia/II) synaptic plasticity. This possibility thus needs to be tested, particularly because of the long silent period expected for Ia afferent synapses disconnected from their peripheral sensory organs. Nonetheless, chronic silencing of Ia afferents with tetrodotoxin applied to the peripheral nerve did not mimic the changes in Ia EPSPs found after injury (Gallego et al., 1979), and Ia axon removal after injury may differ in mechanism from developmental Ia synapse pruning.

In adults, a role was proposed for signaling between ventral microglia and the peripheral immune system through chemokine (C-C motif) ligand 2 (CCL2) activation of its receptor, CCR2 (Rotterman et al., 2019). In global CCR2 KOs, VGLUT1(Ia/II) synapses on dendrites were preserved while synapses on the cell body showed a trend towards preservation that did not reach significance. CCL2 upregulation in axotomized motoneurons was first shown in the facial nucleus (Flugel et al., 2001). CCL2 is also upregulated by sensory afferents in the dorsal root ganglion (Niemi et al., 2013, 2016) and by reactive Schwann cells (Carroll and Frohnert, 1998; Toews et al., 1998; Taskinen and Röyttä, 2000; Subang and Richardson, 2001). At these peripheral locations, CCL2 recruits CCR2-expressing immune cells. Interference with this mechanism affects sensory afferent axon growth in the regenerating nerve and the removal of debris from cut distal axon segments during Wallerian degeneration (reviewed by Zigmond and Echevarria, 2019). CCR2 activation inside the CNS is related to the recruitment of blood-derived immune cells after a variety of injuries or pathologies (Ransohoff, 2009). In the spinal cord, CCL2 exerts a variety of actions, including promoting nociceptive responses in the dorsal horn after nerve injury (Van Steenwinckel et al., 2011), removing damaged myelinated axons after spinal cord injury (Ma et al., 2002; McPhail et al., 2004; Evans et al., 2014) and promoting the breakdown of the blood spinal cord barrier (Echeverry et al., 2011). Infiltration of CCR2 positive immune cells was specifically observed in the ventral horn and only after nerve injuries causing maximal loss of VGLUT1(Ia/II) synapses (Rotterman et al., 2019). Their significance during the removal of ventral Ia axons remains to be fully explored.

## Functional Consequences of the Removal of Ia Afferent Input for Motor Control of Behaviors

The most direct consequence of reduced Ia input on motoneurons is the loss of stretch reflexes. This was first analyzed in a study prompted by the large number of nerve injuries after World War II (Barker and Young, 1947). The goal was to identify differences amongst nerve injuries that could predict better or worse motor function recovery after regeneration. The authors tested the knee stretch reflex in rabbits after nerve crush or transection by measuring the strength of the kick following a tap on the patellar tendon. They found that the stretch reflex recovers (and overshoots) after nerve crush, but never recovers after transection, while muscle force similarly recovers after both injuries. This fits well with data on the Ia-motoneuron synapse reviewed above, but surprisingly the study was largely ignored. The lack of stretch reflexes after recovery from nerve transections was re-discovered in cats years later using electrophysiological methods (Cope and Clark, 1993; Cope et al., 1994) and was also confirmed in rodents (Haftel et al., 2005). The better recovery and even overshoot of stretch reflexes after nerve crush was also replicated in cat experiments (Cope and Clark, 1993; Prather et al., 2011) and agrees with the preservation of VGLUT1(Ia/II) synapses in rats following nerve crush (Schultz et al., 2017).

In contrast, the partial recovery of electrically-evoked Ia EPSPs following nerve transections does not match well with the lack of stretch reflexes after regeneration. The response of motoneurons to naturally evoked stretch synaptic potentials (SSPs) was found to correlate better with reflex function than Ia EPSP responses elicited by electrical stimulation. Thus, while all regenerated motoneurons display Ia EPSPs evoked by electrical afferent volleys in the nerve, many lack SSPs or these are strongly reduced (Haftel et al., 2005; Bullinger et al., 2011). This occurs despite normal stretch afferent responses recorded in dorsal roots entering the spinal cord. In contrast, after nerve crush, SSPs recovered to 75% of their normal size (Prather et al., 2011). One possible explanation is that after nerve transection injuries many Ia afferents fail to reinnervate muscle spindles and although they are recruited in electrically-evoked Ia EPSPs they cannot contribute to stretch-evoked responses. A working model was proposed suggesting a combination of peripheral deficits in spindle innervation and central deficits in Ia synapses and/or their integration in dendrites to fully explain the phenomenon (Alvarez et al., 2010; Bullinger et al., 2011; Vincent et al., 2015).

The stretch reflex is just an easily testable motor behavior of the efficacy of Ia inputs modulating spinal motor output. Its absence, however, implies that ventral horn motor circuitries operate without Ia feedback about muscle lengths and dynamics after regeneration from nerve transections, thus affecting many critical spinal control mechanisms. Accordingly, motor tasks involving high forces and/or rapid and large muscle lengthening (steep slopes) show deficits (Abelew et al., 2000; Maas et al., 2007; Sabatier et al., 2011b; Lyle et al., 2017; Chang et al., 2018). Moreover, the lack of effective Ia inputs in the ventral horn might also affect circuitries like reciprocal inhibition and explain the presence of reciprocal excitation between antagonistic muscles and higher co-contraction and joint stiffness during motor function following regeneration from nerve transections (Sabatier et al., 2011a; Horstman et al., 2019; see Figure 10 in Horstman et al., 2019 for putative circuit mechanisms). Why would this occur after nerve transection but not after nerve crush? Nerve crush preserves continuity in the guiding endoneurial tubes that direct regenerating axons towards the original targets and therefore are characterized by more rapid and specific regeneration compared to the slow and rather poor regeneration specificity after nerve transection (Brushart and Mesulam, 1980; Bodine-Fowler et al., 1997; Valero-Cabré et al., 2004). Peripheral targeting errors must necessarily scramble motor pool organization in the spinal cord and the specific patterns of Ia connections with homonymous and heteronymous motoneurons while avoiding antagonists. This could render Ia connectivity in the ventral horn dysfunctional. Perhaps, evolutionary forces directed the appearance of mechanisms that recognize signals in the periphery correlated with injury severity such that synaptic reorganizations of Ia afferent connections induced by central microglia-neuroinflammation are scaled to the different levels of ambiguity in regeneration specificity in the periphery after different types of nerve injuries.

## Conclusions

The reviewed data fits with a model considering two types of synaptic plasticity after nerve injury. One is a cell-autonomous mechanism that sheds synapses from the cell body (synaptic stripping) and affects GABA/glycine and glutamatergic synapses to different levels in different motoneurons according to modulatory influences from neighboring glial, local neurotrophic factors and the intrinsic susceptibility of different inputs to detachment. A second mechanism is microglia-dependent and induces the retraction from the ventral horn of axon collaterals and synapses originating from axons injured in the peripheral nerve. The degree of axon removal is governed by the type and location of the nerve injury and after removal, there is no recovery of these connections. This mechanism affects the ventral horn collaterals of muscle proprioceptors injured in the periphery, and probably also the intraspinal collaterals of motor axons (Havton and Kellerth, 1990). Thus, after nerve regeneration, the spinal cord ventral horn operates without feedback about muscle length (proprioceptive synapses) or motor output (recurrent motor axon collaterals) causing long-lasting changes in motor function. The extent to which the loss of these inputs represents an undesirable outcome affecting motor function recovery or an adaptive mechanism that optimizes central connections to the vagaries of jumbled connectivity in the periphery after regeneration is unknown and currently under investigation.

## Author Contributions

FA wrote the manuscript and designed the figures. TR compiled literature regarding synaptic stripping, microglia and provided the primary data on Ia afferent and motor axon plasticity. EA compiled the literature on KCC2 and inhibitory synaptic function after axotomy and provided some of the data on synaptic stripping. AL provided some of the primary data on synaptic stripping after preventing microglia reactions. TR, EA, and AL revised the manuscript. TC and AE were involved in the genesis of some ideas in this review.

## Conflict of Interest

The authors declare that the research was conducted in the absence of any commercial or financial relationships that could be construed as a potential conflict of interest.
